# Antiproliferation Effects of Marine-Sponge-Derived Methanol Extract of *Theonella swinhoei* in Oral Cancer Cells In Vitro

**DOI:** 10.3390/antiox11101982

**Published:** 2022-10-04

**Authors:** Jun-Ping Shiau, Ya-Ting Chuang, Jen-Yang Tang, Shu-Rong Chen, Ming-Feng Hou, Jiiang-Huei Jeng, Yuan-Bin Cheng, Hsueh-Wei Chang

**Affiliations:** 1 Division of Breast Oncology and Surgery, Department of Surgery, Kaohsiung Medical University Hospital, Kaohsiung Medical University, Kaohsiung 80708, Taiwan; 2 Graduate Institute of Medicine, College of Medicine, Kaohsiung Medical University, Kaohsiung 80708, Taiwan; 3 School of Post-Baccalaureate Medicine, Kaohsiung Medical University, Kaohsiung 80708, Taiwan; 4 Department of Radiation Oncology, Kaohsiung Medical University Hospital, Kaoshiung Medical University, Kaohsiung 80708, Taiwan; 5 Department of Marine Biotechnology and Resources, National Sun Yat-sen University, Kaohsiung 80424, Taiwan; 6 Department of Biomedical Science and Environmental Biology, College of Life Science, Kaohsiung Medical University, Kaohsiung 80708, Taiwan; 7 School of Dentistry, College of Dental Medicine, Kaohsiung Medical University, Kaohsiung 80708, Taiwan; 8 Department of Dentistry, Kaohsiung Medical University Hospital, Kaohsiung 80708, Taiwan; 9 Department of Dentistry, National Taiwan University Hospital, Taipei 100225, Taiwan; 10 Center for Cancer Research, Kaohsiung Medical University, Kaohsiung 80708, Taiwan

**Keywords:** marine sponge, oral cancer, oxidative stress, natural product

## Abstract

The purpose of this study aimed to assess the antiproliferation effects of methanol extract of *T. swinhoei* (METS) and explore the detailed responses of oral cancer cells compared to normal cells. METS effectively inhibits the cell proliferation of oral cancer cells but does not affect normal cell viability, exhibiting preferential antiproliferation function. METS exerted more subG1 accumulation, apoptosis induction, cellular and mitochondrial oxidative stress, and DNA damage than normal cells, reverted by oxidative stress inhibitor *N*-acetylcysteine. This METS-caused oxidative stress was validated to attribute to the downregulation of glutathione. METS activated both extrinsic and intrinsic caspases. DNA double-strand breaks (γH2AX) and oxidative DNA damage (8-hydroxy-2-deoxyguanosine) were stimulated by METS. Therefore, for the first time, this investigation shed light on exploring the functions and responses of preferential antiproliferation of METS in oral cancer cells.

## 1. Introduction

Oral cancer patients show high morbidity and mortality [1] and show high incidence worldwide [2]. Surgery, radiation, and chemotherapy are common clinical therapies for oral cancer, but chemoradiation occasionally generates side effects [3]. It is necessary to identify new anticancer drugs for oral cancer treatment.

Marine natural products are rich resources for anti-cancer agents [4,5,6,7]. Marine sponges are the largest biomass in the ocean and contain diverse bioactive compounds [8,9,10,11] for cancer, inflammation, viral, and antibiotic treatments [10,12]. Sponge *Niphates* sp.-derived proteinaceous factions show antibacterial properties [13]. *Spongosorites halichondriodes* extracts show antiinflammation effects [14]. Accordingly, the anticancer effects on several kinds of marine sponges warrant detailed re-examination.

Several extraction studies recently isolated several pure compounds from the marine sponge *Theonella swinhoei* (*T. swinhoei*) [15,16,17,18,19,20,21,22,23]. However, most studies [17,23] focused on bioactive compound isolations of the marine sponge *T. swinhoei*. They provided IC_50_ values for certain cancer cell lines such as liver (HepG2) [18], colon (HCT-16) [19], lymphoma (P388) [20], and breast (MCF7 and MDA-MB-231) [22,23] without investigating its antiproliferation mechanism. However, the antiproliferation studies of *Theonella* natural products were not reported in oral cancer cells and evaluated for their safety in normal cells.

Crude extracts of natural products contain several bioactive compounds. Different compounds may modulate different targets, providing the benefits of the potential for multiple targeting of cancer [24]. Such multiple-target strategy of natural products has been applied to inflammation [25] and cancer [26] treatments. Notably, sponges contain natural products such as terpenoids, glycosides, phenols, fatty acids, peptides, sterols, and others [27]. Accordingly, the anticancer effects of *Theonella* crude extract warrants a detailed investigation of oral cancer cells.

This investigation evaluates the antiproliferation effects of methanol extracts of *T. swinhoei* (METS) and explores the antiproliferation mechanism of oral cancer cells. Several oral cancer and normal cells were included to examine their proliferation, oxidative stress, apoptosis, and DNA damage responses.

## 2. Materials and Methods

### 2.1. METS Preparation

The sponge *Theonella swinhoei* was collected by scuba diving in Orchid Island, Taitung County, Taiwan, in April 2011. A voucher specimen (OISP-1) was deposited at the Department of Marine Biotechnology and Resources, National Sun Yat-sen University, Kaohsiung, Taiwan. The animal material was extracted by ethanol thrice to provide a crude extract (469.0 g). This extract was aliquoted into ethyl-acetate-soluble and water-soluble fractions, and the former portion was further partitioned between 75% methanol (aq) and hexanes. The 75% methanol soluble layer (43.9 g) of *T. swinhoei* was termed METS.

### 2.2. HPLC Analysis of METS

The Shimadzu HPLC system contains an LC-40D solvent delivery module, a DGU-405 degassing unit, an SPD-M40A photo diode array detector, a CTO-40S column oven, and a CBM-40 controller. The Phenomenex (Torrance, CA, USA) Luna 5 µ C18(2) 100 A analytical column was chosen for separation. The chromatography conditions were shown as follows. Solution A: 0.1% trifluoroacetic acid (aq); solution B: MeOH; flow rate: 1.0 mL/min; 0 min: 85% solution B, 0–10 min: 85% to 95% solution B, 10–20 min: 95% solution B, 20–30 min: 95% to 100% solution B. Injection weight: 10 µg.

### 2.3. Cell Cultures and Reagents

Oral cancer cell lines such as tongue-derived CAL 27 (ATCC, Manassas, VA, USA) and Gingiva-derived Ca9-22 (HSRRB, Ibaraki, Osaka, Japan) were used. To assess the drug safety of METS, a non-malignant cell line derived from gingival epithelial Smulow-Glickman (S-G) was chosen as normal control cells [28,29,30]. The culture medium was prepared by mixing DMEM with F12 (3:2) (Gibco, Grand Island, NY, USA), 10% fetal bovine serum, and P/S antibiotics [31]. Cells were seeded at 4 × 10^4^/well for a 12-well plate for growth overnight and subjected to drug treatment for flow cytometry experiments.

*N*-acetylcysteine (NAC; 10 mM, 1 h pretreatment) (Sigma-Aldrich, St. Louis, MO, USA) [32,33,34] was used to check the influence of oxidative stress in METS experiments.

### 2.4. Cell Viability Assay

MTS reagent (Promega Corporation, Madison, WI, USA), a tetrazolium dye, was used to detect cell viability. Ca9-22, CAL 27, and S-G cells were plated at 4, 4, and 6 × 10^3^/well for a 96-well plate. Subsequently, cells were incubated overnight. Finally, cells were then treated with drugs for 24 h. Finally, MTS reagents were added and incubated for 1 h before ELISA reader detection (490 nm) [35].

### 2.5. Cytometric Cell Cycle Assay

Cell cycle phases are proportional to the DNA content, which was stained using 1 μg/mL of 7-aminoactinmycin D (7AAD) (Biotium, Hayward, CA, USA) for 30 min for 75% ethanol fixed cells [36]. DNA content was assessed using a flow cytometer (Guava easyCyte, Luminex, TX, USA), and cell cycle phases were analyzed using Flow Jo 10 software (Becton-Dickinson; Franklin Lakes, NJ, USA).

### 2.6. Cytometric Apoptosis Assay (Annexin V/7AAD)

Phosphatidylserine located at the outer plasma membrane of apoptotic cells is proportional to the annexin V detection, which is stained by annexin V-FITC/7AAD (1:1000/1 μg/mL) [37] (Strong Biotech, Taipei, Taiwan) for 1 h. Guava easyCyte flow cytometer assessed these intensities. The counts for annexin V (+)/7AAD (+ or −) regions were classified as apoptotic (+) cells.

### 2.7. Peptide-Based Apoptosis Assay (Caspase 3/7)

The caspases-Glo^®^ *3/7* luminescent kit (Promega; Madison, WI, USA) utilizes proluminescent peptide substrates to detect caspase 3/7 activity [38]. When the caspase 3/7 was activated in apoptotic cells, the proluminescent peptide substrates were converted to luminescent signals monitored by a luminometer (Berthold Technologies GmbH & Co., Bad Wildbad, Germany). The caspase 3/7 intensities were calibrated by adjusting the respective cell viabilities.

### 2.8. Cytometric Apoptosis Assay (Caspases 3, 8, and 9)

Activities of caspases 3, 8, and 9 are proportional to the staining intensities by incubating their specific peptide substrates (PhiPhiLux-G1D2, CaspaLux8-L1D2, and CaspaLux9-M1D2) (OncoImmunin; Gaithersburg, MD, USA) according to user’s instruction [39,40]. The caspases 3, 8, and 9 activated reactions can generate green fluorescence after peptide cleavage, i.e., caspase activation. The Guava easyCyte flow cytometer assessed these intensities. Their positive (+) region counts were classified as caspases 3, 8, and 9 (+) cells.

### 2.9. Cytometric Reactive Oxygen Species (ROS), Mitochondrial Superoxide (MitoSOX), and Glutathione (GSH) Assays

Levels of ROS, MitoSOX, and GSH are proportional to the staining intensities by incubating their specific dyes such as 2′,7′-dichlorodihydrofluorescein diacetate (H2DCFDA) (Sigma-Aldrich, St. Louis, MO, USA) [31], MitoSOX™ Red, and 5-chloromethylfluorescein diacetate (CMF-DA) (Thermo Fisher Scientific, Carlsbad, CA, USA) [41] in the darkness conditions (10 μM/30 min, 50 nM/30 min, and 5 μM/20 min at 37 °C). The Guava easyCyte flow cytometer assessed these intensities. Their counts for (+) regions were classified as ROS, MitoSOX, and GSH (+) cells.

### 2.10. Cytometric γH2AX/7AAD and 8-Hydroxy-2-deoxyguanosine (8-OHdG) Assays

Levels of γH2AX and 8-OHdG [42] are proportional to the fluorescent intensities generated by antibody detection to the 75% ethanol fixed cells. Primary antibodies for γH2AX [42] (Santa Cruz Biotechnology, Santa Cruz, CA, USA) and FITC-8-OHdG antibody (Santa Cruz Biotechnology, Santa Cruz, CA, USA) were used to incubate with these fixed cells at 4 °C, 1 h. Notably, secondary antibody (Cell Signaling Technology, Danvers, MA, USA) and 7AAD (5 μg/mL, 30 min) were further applied for γH2AX detection. The Guava easyCyte flow cytometer assessed these intensities. Their counts for (+) regions were classified as γH2AX and 8-OHdG (+) cells.

### 2.11. Statistical Analysis

The one-way ANOVA analysis and post hoc test (JMP software, SAS Institute Inc., Cary, NC, USA) provides the connecting letters for determining the significant difference for multiple comparison. Connecting letters without an overlap indicate a significant difference, while it differs non-significantly when the connecting letters are overlapping. Examples were mentioned in the figure legend to explain significance.

## 3. Results

### 3.1. HPLC Analysis of METS

**Figure 1:**
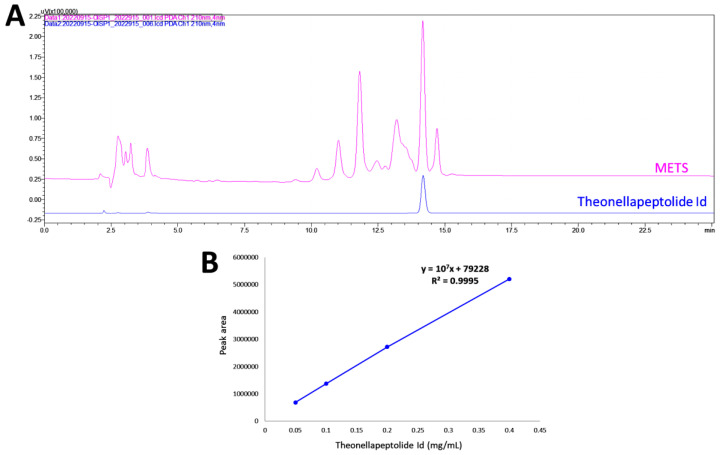
Fingerprint profile. (**A**) Fingerprint profiles of METS (pink, upper line) and the theonellapeptolide 1d (blue, lower line). (**B**) The calibration curve of theonellapeptolide 1d (*n* = 3).

HPLC-PDA fingerprint profiles of METS and the isolated major compound, the-onellapeptolide 1d, were provided (Figure 1A). The retention time of theonellapeptolide 1d was found at 14.205 min, which overlapped the major peak of METS. The linear equations (*y* = 10^7^*x* − 79228, *R*^2^ = 0.9995) of theonellapeptolide 1d was deduced by the HPLC peak area in four different concentrations (Figure 1B). In METS, theonellapeptolide 1d accounts for 21.0% of the whole amount.

### 3.2. METS Causes More Antiproliferation to Oral Cancer Cells than Normal Cells

**Figure 2:**
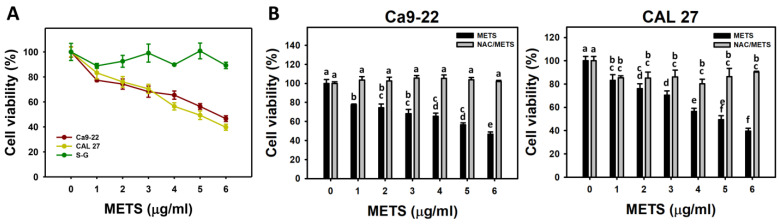
METS causes antiproliferation of oral cancer cells. (**A**) Cell viability. Cells were treated with METS for 24 h. Oral cancer (Ca9-22 and CAL 27) and normal cells (S-G) were included. (**B**) NAC response on cell viability of METS treatment. Untreated control and NAC pretreatment were followed by posttreatment of vehicle control (0 μg/mL) and METS (1, 2 ,3, 4, 5, and 6 μg/mL) for 24 h, i.e., control, NAC, METS, and NAC/METS, respectively. Data = mean ± SD (*n* = 3). Data showing non-overlapping lower-case letters have significant results (*p* < 0.05) as analyzed using the one-way ANOVA analysis and post hoc test. In the example of Figure 1B (Ca9-22 cells), the lower-case letters for METS at 0, 1, 4, and 6 μg/mL are “a, b, cd, and e”, revealing significant differences between each other. By contrast, the lower-case letters for METS at 1, 2, and 3 μg/mL are “b, bc, and bc”, indicating non-significant differences since they overlap with the same letter “b”.

METS decreased the cell viability for oral cancer cells (Ca9-22 and CAL 27) (Figure 2A). The drug safety of METS was examined using the viability of normal cells (S-G). These METS-treated normal cells exhibited healthy viability like the control. Additionally, NAC, a ROS removal agent, was chosen to test the contribution of ROS in antiproliferation caused by METS. Under different concentrations of METS, NAC alleviated the METS-promoted antiproliferation against oral cancer cells (Figure 2B), revealing the oxidative-stress-dependent action of METS.

### 3.3. METS Causes More subG1 Accumulation to Oral Cancer Cells than Normal Cells

**Figure 3:**
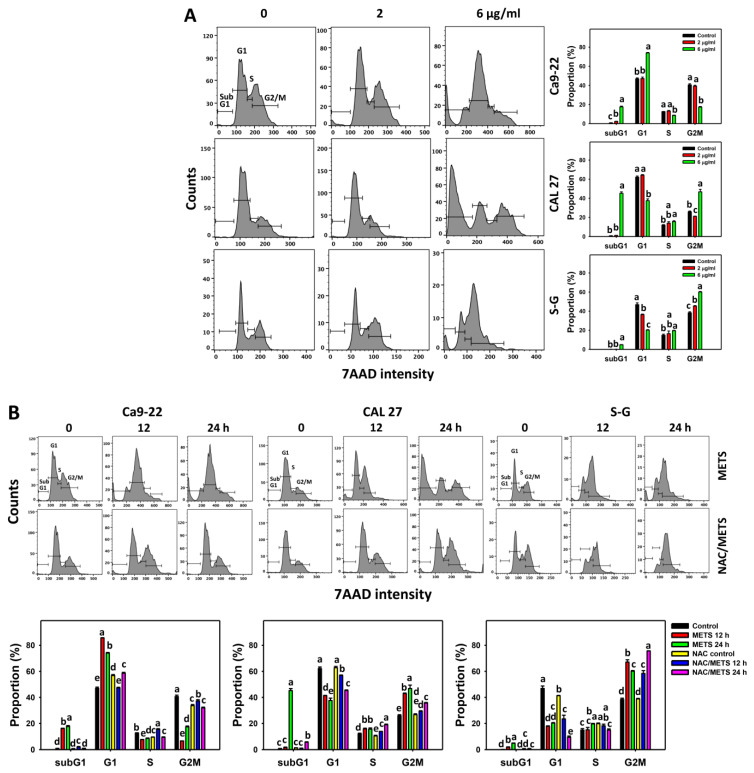
METS causes subG1 accumulation of oral cancer cells. (**A**) Cell cycle changes. Cells were treated with METS for 24 h. Oral cancer and normal cells (S-G) were included. (**B**) NAC response on cell cycle of METS treatment. Untreated control and NAC pretreatment were followed by posttreatment of vehicle control and METS (6 μg/mL) for 0, 12, and 24 h, i.e., control, NAC, METS, and NAC/METS, respectively. Data = mean ± SD (*n* = 3). Data showing non-overlapping lower-case letters have significant results (*p* < 0.05) as analyzed by the one-way ANOVA analysis and post hoc test.

The subG1 events of histograms were used to measure primarily apoptosis. METS caused the accumulation of subG1 events in oral cancer cells (Ca9-22 and CAL 27) under dose and time course experiments (Figure 3A,B). CAL 27 cells showed a higher extent of subG1 events than Ca9-22 cells. In contrast, METS exhibited lower subG1 events in normal S-G cells than oral cancer cells. Accordingly, METS exerts a greater extent of subG1 in oral cancer than in normal cells.

The G1 events were increased, and G2/M events were decreased at 6 μg/mL of METS for Ca9-22 cells. By contrast, the G1 events were reduced, and G2/M events were increased at 6 μg/mL of METS for CAL 27 and S-G cells. Accordingly, METS exerts differential cell cycle disturbance for various cell lines.

Additionally, NAC was used to test the contribution of ROS in cell cycle disturbance caused by METS. NAC inhibited subG1 increment of METS-treated oral cancer cells (Figure 3B), revealing that oxidative stress modulated METS-triggered subG1 increment. NAC inhibited G1 increment and G2/M decrement of METS-treated Ca9-22 cells. In contrast, NAC inhibited G1 decrement and G2/M increment of METS-treated CAL 27 cells. For normal S-G cells, NAC enhanced G1 decrement and G2/M increment at 6 μg/mL of 24 h METS treatment.

### 3.4. METS Causes More Annexin V-Based Apoptosis to Oral Cancer Cells than Normal Cells

**Figure 4:**
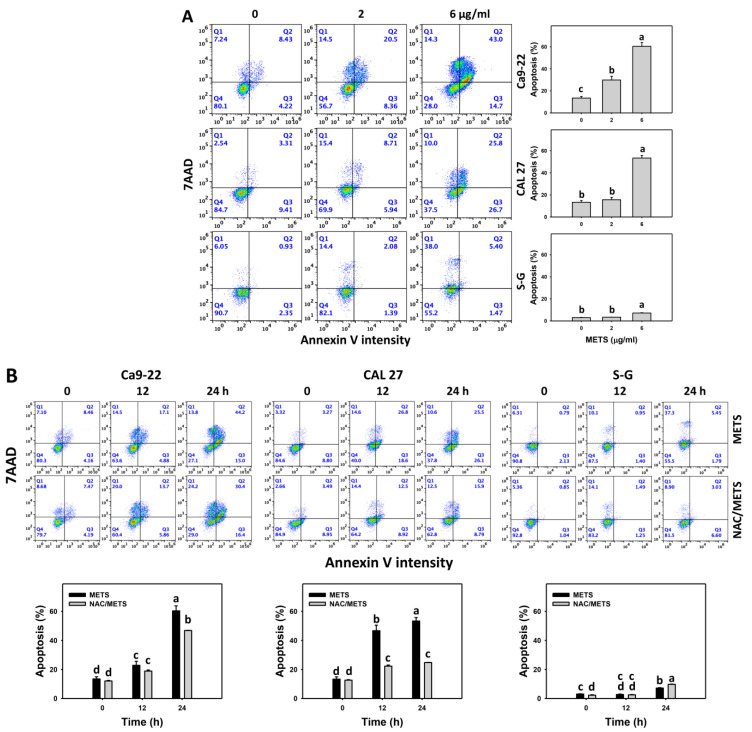
METS causes annexin-V-assessed apoptosis in oral cancer cells. (**A**) Annexin V histogram changes. Cells were treated with METS for 24 h. Oral cancer and normal cells (S-G) were included. Annexin V (+)/7AAD (+ or −) events were counted for apoptosis (%). (**B**) NAC response on annexin V histogram changes of METS treatment. Untreated control and NAC pretreatment were followed by posttreatment of vehicle control and METS (6 μg/mL) for 0, 12, and 24 h, i.e., control, NAC, METS, and NAC/METS, respectively. Data = means ± SD (*n* = 3). Data showing non-overlapping lower-case letters have significant results (*p* < 0.05) as analyzed by the one-way ANOVA analysis and post hoc test.

SubG1 increment is an apoptosis-like change, and it needs further validation for apoptosis. The annexin V (+) events of histograms were used to measure apoptosis. Annexin V (+) events were dose- and time-dependently increased by METS in oral cancer cells (Ca9-22 and CAL 27) (Figure 4). However, it indicated lower annexin (+) events in normal S-G cells than in oral cancer cells by METS treatment. Accordingly, METS exerts a greater extent of apoptosis induction in oral cancer cells than normal cells.

Additionally, NAC was used to test the contribution of ROS in annexin V increment caused by METS. NAC inhibited annexin V increment of METS-treated oral cancer cells (Figure 4B), particularly for 24 h. It reveals that oxidative stress modulated METS-triggered apoptosis.

### 3.5. METS Causes More Caspase 3 and 3/7 Activations to Oral Cancer Cells than Normal Cells

**Figure 5:**
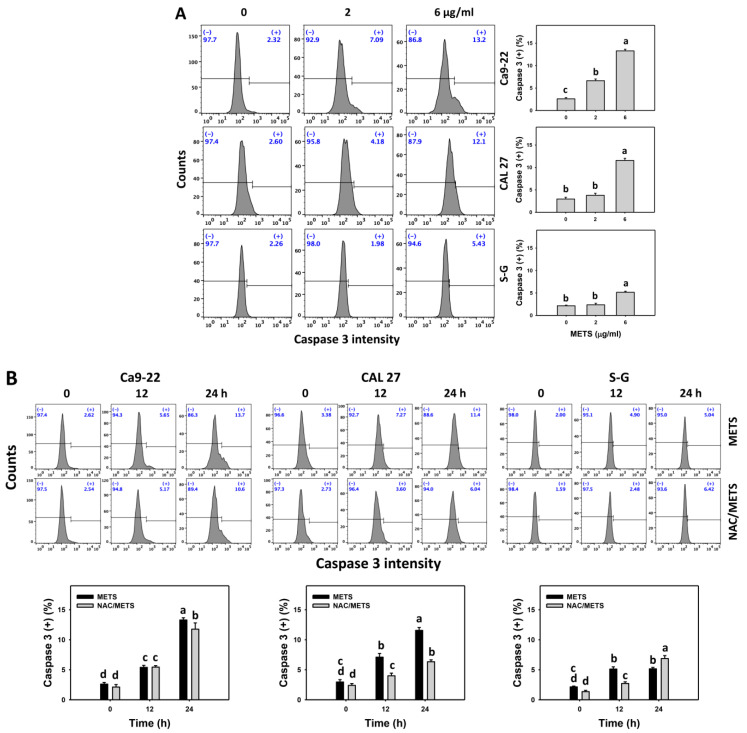

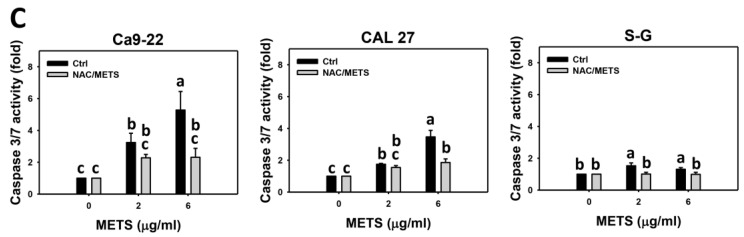
METS leads to caspase 3 and 3/7 activations in oral cancer cells. (**A**) Caspase 3 histogram changes. Cells were treated with METS for 24 h. Oral cancer and normal cells (S-G) were included. (+) indicates a high intensity of caspase 3. (**B**,**C**). NAC response on caspase 3 and 3/7 activations. For caspase 3 assay, untreated control and NAC pretreatment were followed by posttreatment of vehicle control and METS (6 μg/mL) for 0, 12, and 24 h, i.e., control, NAC, METS, and NAC/METS, respectively. Except for 24 h, other conditions of caspase 3/7 assays were the same as caspase 3 assay. Data = mean ± SD (*n* = 3). Data showing non-overlapping lower-case letters have significant results (*p* < 0.05) as analyzed by the one-way ANOVA analysis and post hoc test.

Caspase 3 activation was monitored by flow cytometry and luminescence detection. For flow cytometry, the caspase 3 (+) events of histograms were used to measure apoptosis. Caspase 3 (+) events were dose- and time-dependently upregulated by METS in oral cancer cells (Ca9-22 and CAL 27) (Figure 5A,C). However, it showed lower caspase 3 (+) events in normal S-G cells than oral cancer cells by METS treatment. For the luminescent assay, caspase 3/7 activities were dose-responsively increased in oral cancer cells but not in normal cells (Figure 5C). Accordingly, METS exert more caspase 3 and 3/7 activations in oral cancer cells than in normal cells.

Additionally, NAC was used to test the contribution of ROS in caspase 3 and 3/7 activation caused by METS. NAC moderately inhibited caspase 3 activations of Ca9-22 cells at 24 h METS treatment (Figure 5B). NAC dramatically inhibited caspase 3 activations of CAL 27 cells at 12 and 24 h METS treatment. NAC also moderately inhibited 3/7 activations of Ca9-22 and CAL 27 cells at 6 μg/mL of 24 h METS treatment (Figure 5B,C). In contrast, normal S-G cells showed low changes in caspase 3 and 3/7 activities. These results reveal that oxidative stress modulated METS-triggered caspase 3 and 3/7 activations.

### 3.6. METS Causes More Caspases 8 and 9 Activations to Oral Cancer Cells than Normal Cells

**Figure 6:**
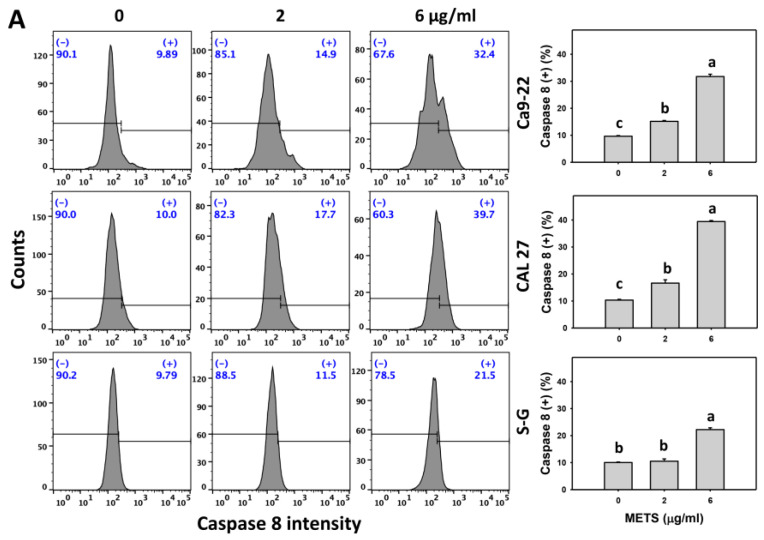

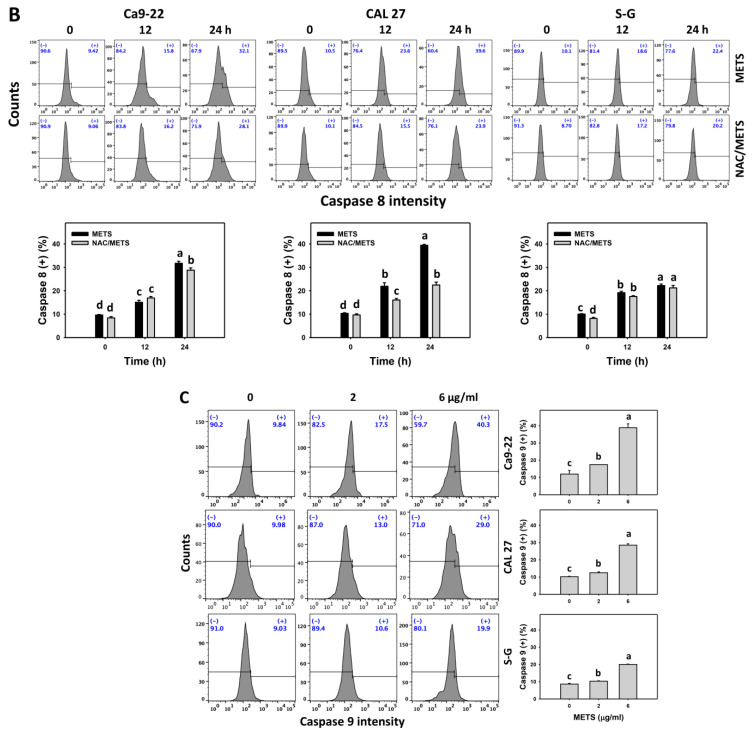

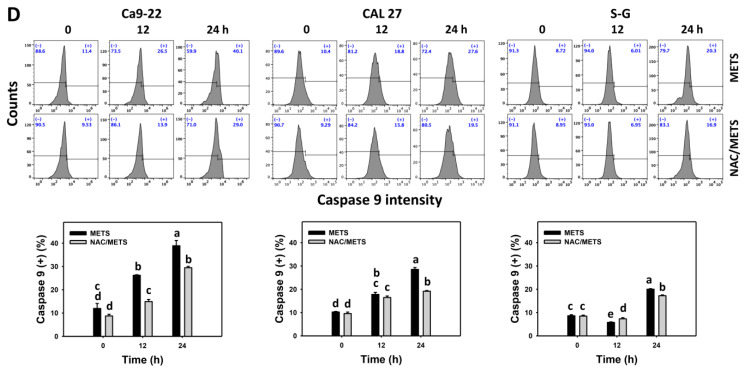
METS leads to Cas 8 and 9 activations in oral cancer cells. (**A**,**C**) Caspases 8 and 9 histogram changes. Cells were treated with METS for 24 h. Oral cancer (Ca9-22 and CAL 27) and normal cells (S-G) were included. (+) indicates a high intensity of caspases 8 and 9. (**B**,**D**). NAC response on caspases 8 and 9 activations. Untreated control and NAC pretreatment were followed by posttreatment of vehicle control and METS (2 and 6 μg/mL) for 0, 12, and 24 h, i.e., control, NAC, METS, and NAC/METS, respectively. Data = mean ± SD (*n* = 3). Data showing non-overlapping lower-case letters show significant results (*p* < 0.05) as analyzed by the one-way ANOVA analysis and post hoc test.

Caspase 8 and 9 activations were monitored by flow cytometry. The caspase 8 and 9 (+) events of histograms were used to measure the apoptosis. Caspase 8 and 9 (+) events were dose- and time-dependently increased by METS in oral cancer cells (Ca9-22 and CAL 27) (Figure 6A,C). However, it showed lower caspase 8 and 9 (+) events in normal S-G cells than oral cancer cells by METS treatment. Accordingly, METS exert more caspase 8 and 9 activations in oral cancer cells than in normal cells. Additionally, NAC inhibited caspase 8 and 9 activations of METS-treated oral cancer cells (Figure 6B,D), revealing that oxidative stress modulated METS-triggered apoptotic signaling.

### 3.7. METS Causes More ROS and MitoSOX but Less GSH Generations to Oral Cancer Cells than Normal Cells

**Figure 7:**
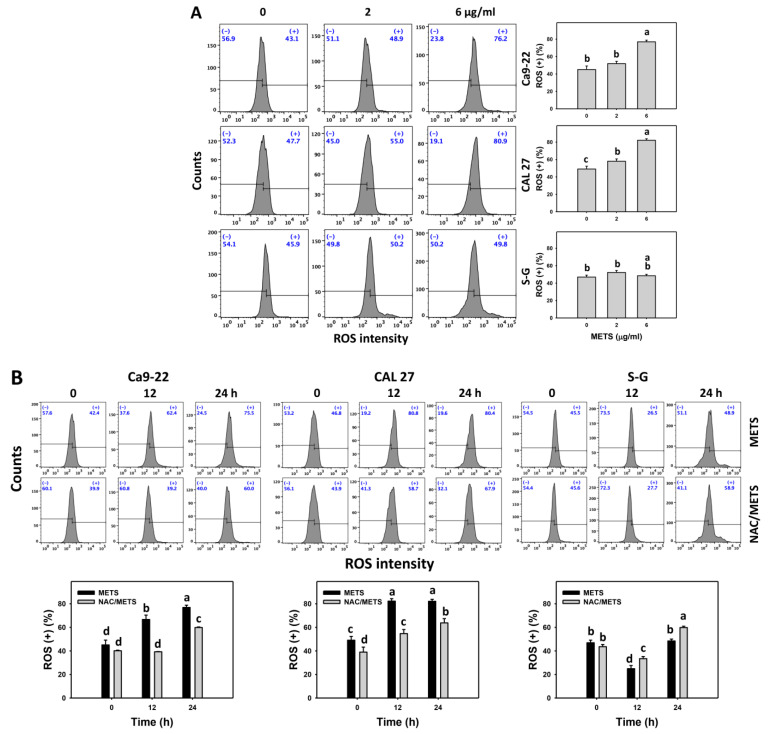

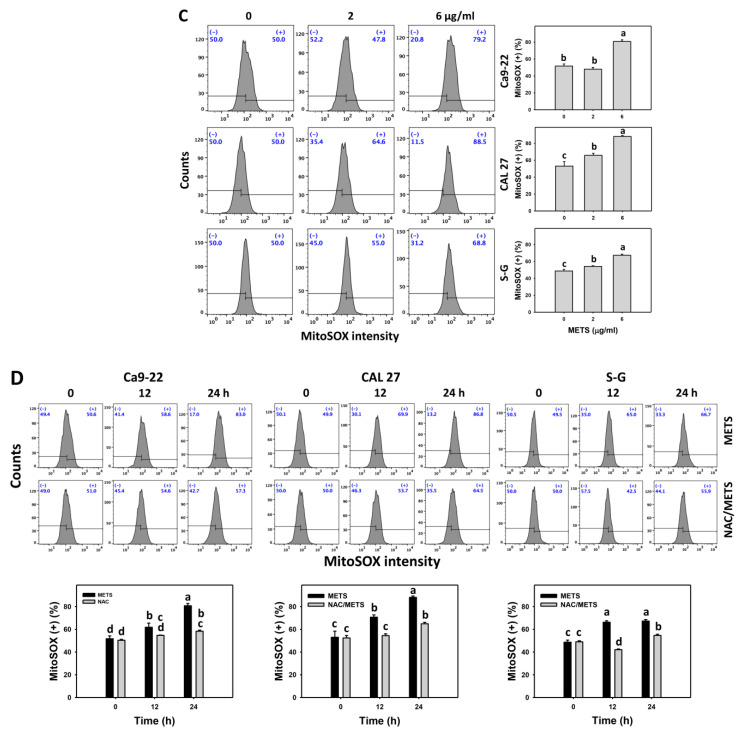
METS improves ROS and MitoSOX levels of oral cancer cells. (**A**,**C**) ROS and MitoSOX histogram changes. Cells were treated with METS for 24 h. Oral cancer and normal cells (S-G) were included. (+) indicates the high intensity of ROS and MitoSOX. (**B**,**D**) NAC response on ROS and MitoSOX levels. Untreated control and NAC pretreatment were followed by posttreatment of vehicle control and METS (6 μg/mL) for 0, 12, and 24 h, i.e., control, NAC, METS, and NAC/METS, respectively. Data = mean ± SD (*n* = 3). Data showing non-overlapping lower-case letters provide significant results (*p* < 0.05) as analyzed by the one-way ANOVA analysis and post hoc test.

The ROS and MitoSOX (+) events of histograms were used to measure oxidative stress. In general, ROS and MitoSOX (+) events were dose- and time-dependently increased by METS in oral cancer cells (Ca9-22 and CAL 27) (Figure 7). However, it showed lower ROS and MitoSOX (+) events in normal S-G cells than in oral cancer cells by METS treatment. Accordingly, METS exerts more ROS and MitoSOX in oral cancer cells than in normal cells. Additionally, NAC inhibited ROS and MitoSOX increment of METS-treated oral cancer cells (Figure 7B), revealing that oxidative stress participated in METS-triggered ROS and MitoSOX generation.

**Figure 8:**
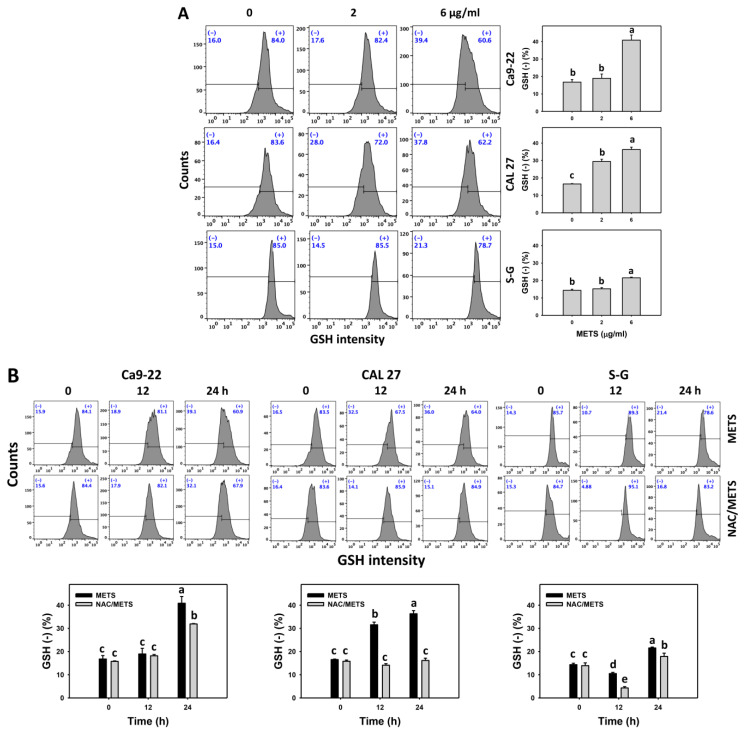
METS depletes the GSH level of oral cancer cells. (**A**) GSH histogram changes. Cells were treated with METS for 24 h. Oral cancer and normal cells (S-G) were included. (+) indicates the high intensity of GSH. (**B**) NAC response on GSH levels. Untreated control and NAC pretreatment were followed by posttreatment of vehicle control and METS (6 μg/mL) for 0, 12, and 24 h, i.e., control, NAC, METS, and NAC/METS, respectively. Data = mean ± SD (*n* = 3). Data marked showing non-overlapping lower-case letters have significant results (*p* < 0.05) as analyzed by the one-way ANOVA analysis and post hoc test.

Moreover, GSH (+) events were decreased by METS in oral cancer cells (Ca9-22 and CAL 27) in dose and time course experiments (Figure 8). However, it showed higher GSH (+) events in normal S-G cells than in oral cancer cells by METS treatment. Accordingly, METS exerts less GSH (+) in oral cancer cells than in normal cells. Additionally, NAC inhibited GSH (+) depletion of METS-treated oral cancer cells (Figure 8B), revealing that oxidative stress participated in METS-triggered GSH depletion.

### 3.8. METS Causes More DNA Damage to Oral Cancer Cells than Normal Cells

**Figure 9:**
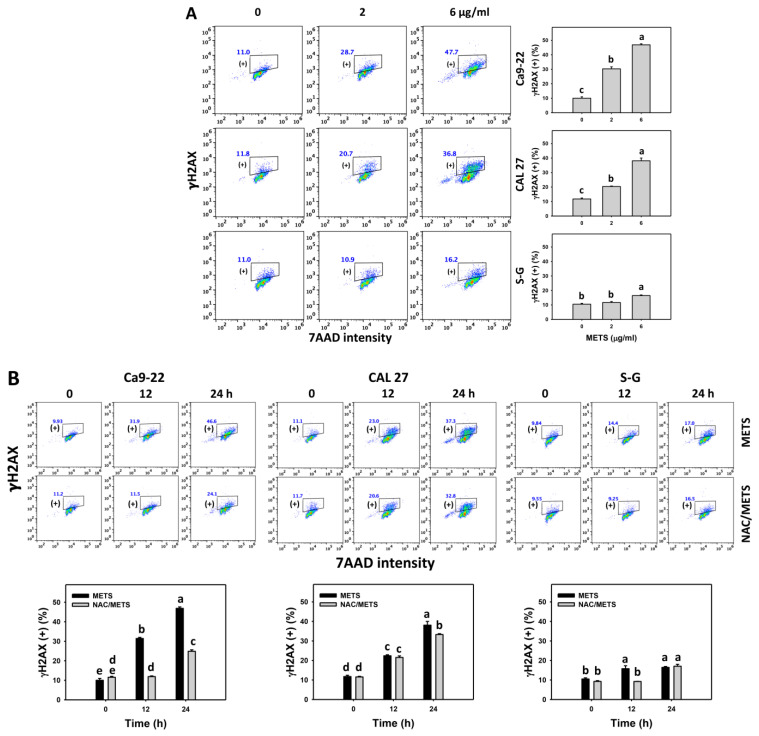
METS enhances the γH2AX level of oral cancer cells. (**A**) γH2AX histogram changes. Cells were treated with METS for 24 h. Oral cancer and normal cells (S-G) were included. (+) indicates the high intensity of γH2AX. (**B**) NAC response on γH2AX levels. Untreated control and NAC pretreatment were followed by posttreatment of vehicle control and METS (6 μg/mL) for 0, 12, and 24 h, i.e., control, NAC, METS, and NAC/METS, respectively. Data = mean ± SD (*n* = 3). Data showing non-overlapping lower-case letters have significant results (*p* < 0.05) as analyzed by the one-way ANOVA analysis and post hoc test.

**Figure 10:**
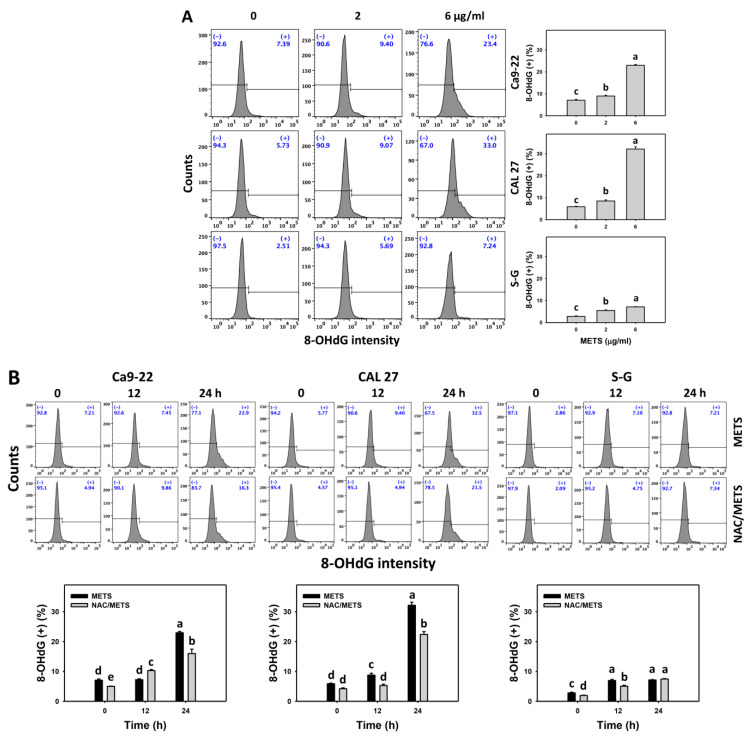
METS enhances the 8-OHdG level of oral cancer cells. (**A**) 8-OHdG histogram changes. Cells were treated with METS for 24 h. Oral cancer and normal cells (S-G) were included. (+) indicates the high intensity of 8-OHdG. (**B**) NAC response on 8-OHdG levels. Untreated control and NAC pretreatment were followed by posttreatment of vehicle control and METS (6 μg/mL) for 0, 12, and 24 h, i.e., control, NAC, METS, and NAC/METS, respectively. Data = mean ± SD (*n* = 3). Data showing non-overlapping lower-case letters have significant results (*p* < 0.05) as analyzed by the one-way ANOVA analysis and post hoc test.

The γH2AX and 8-OHdG (+) events of histograms were used to measure DNA damage. γH2AX and 8-OHdG (+) events were dose- and time-dependently increased by METS in oral cancer cells (Ca9-22 and CAL 27) (Figure 9A and Figure 10A). However, it showed lower γH2AX and 8-OHdG (+) events in normal S-G cells than in oral cancer cells by METS treatment. Accordingly, METS exerts more γH2AX and 8-OHdG in oral cancer cells than in normal cells. Additionally, NAC inhibited γH2AX and 8-OHdG increment of METS-treated oral cancer cells (Figure 9B and Figure 10B), particularly for 24 h. These results reveal that oxidative stress was involved in METS-triggered γH2AX and 8-OHdG generation.

## 4. Discussion

Several kinds of marine sponge extracts have demonstrated anticancer [43] effects. However, the impacts of antiproliferation of the marine sponge *Theonella* extract (METS) on oral cancer cells were rarely investigated. Moreover, most marine sponge extract studies reported only on the cytotoxicity of some cancer cells but did not consider the drug response to normal cells. These studies for marine sponge extracts lack a detailed investigation of their acting mechanism. The present study demonstrated the preferential antiproliferation to oral cancer cells but only revealed the low cytotoxic effect on normal cells. The exact impacts of METS treatments on oral cancer cells were discussed.

### 4.1. Comparison of Antiproliferation Effects of Several Marine Sponge Extracts on Cancer Cells

Several marine sponge extracts reported antiproliferation against cancer cells. For example, methanol/CH_2_Cl extracts of two kinds of *Theonella* sp. show IC_50_ values of 4 and 10 μg/mL for liver cancer cells (Hep3B) at the 24 h CCK-8 assay [44]. Methanol/CH_2_Cl extracts of *Theonella* sp. and *Haliclona* sp. show IC_50_ values of 0.5 and 1 μg/mL for colon cancer cells (HT29) at the 72 h CCK-8 assay [44]. The 70% ethanol extract of *Dysidea avara* shows IC_50_ values of 7.17, 9.55, 20.88, and 25.15 μg/mL for leukemia (K562), myeloma (KMS-12PE and A375), and lung (K549) cancer cells at a 48 h MTT assay [45].

In the present assessment, METS shows IC_50_ values of 4.5 and 5 μg/mL in oral cancer cells (Ca9-22 and CAL 27) at a 24 h MTS assay. In contrast, normal cells (S-G) only show no change in viability. Moreover, this antiproliferation was alleviated by ROS inhibitor (NAC) pretreatment. Accordingly, these results suggest that METS possesses ROS-dependent preferential antiproliferation to oral cancer cells but causes little cell death in normal cells. Since METS is the crude extract from the marine sponge, it shows a high drug sensitivity (IC_50_ 4.5 μg/mL) for oral cancer treatment; however, the in vivo antioral cancer effects of METS were not investigated. The in vivo effect of METS warrants a detailed evaluation that may improve the drug progression of its clinical treatment to oral cancer. Moreover, combining natural products with clinical drugs can improve oral cancer therapy [46,47]. Combined treatment may sensitize cancer cells to clinical medications and reduce their potential adverse effects. It warrants a detailed assessment of future combined treatment, including METS and other clinical drugs.

### 4.2. METS Exhibits Preferential Generation of Oxidative Stress to Oral Cancer Cells

Cancer cells exhibit a higher extent of basal oxidative stress than normal cells [42,48,49,50,51]. Elevated oxidative stress may overload cancer cells and cause cell death but tolerate normal cells to maintain cell survival. Similarly, METS also demonstrated oxidative stress inductions, such as upregulation of ROS and MitoSOX in oral cancer cells more than in normal cells (Figure 7). These oxidative stress inductions were suppressed by NAC, suggesting that MET is an oxidative modulating agent. Accordingly, METS showed preferential induction of oxidative stress in oral cancer but not in normal cells.

Moreover, redox homeostasis is balanced by cellular antioxidants and oxidative stress [52,53]. GSH can alleviate cellular oxidative stress [54]. Some drugs suppress endogenous antioxidants and induce oxidative stress. For instance, amygdalin decreases the GSH level and is associated with oxidative stress induction of breast cancer cells [55]. Similarly, METS downregulate GSH levels in oral cancer cells (Figure 8), accompanied by oxidative stress generation (Figure 7). After METS treatment, the GSH level of oral cancer cells is lower than normal cells. Therefore, METS-induced oxidative stress may partly be attributed to GSH depletion.

### 4.3. METS Preferentially Provokes Apoptosis in Oral Cancer Cells

Oxidative stress elevation is an anticancer strategy promoting apoptosis [49,56]. Marine sponge extracts promote apoptosis in cancer cells [57]. For example, petroleum ether extract of *Negombata magnifica* induces apoptosis in terms of DNA ladder assay in liver cancer cells [57]. Ethyl acetate extract of *Hyalella cribriformis* activates caspase 3 in rhabdomyosarcoma cells [58].

Similarly, METS causes a dramatic elevation of subG1 (Figure 3), and it also was associated with apoptosis inductions as detected by annexin V staining (Figure 4). Moreover, apoptosis-related caspase signaling, such as caspases 8, 9, and 3, were activated by METS of oral cancer cells but displayed a small activation in normal cells. These results indicate that METS preferentially causes apoptosis in oral cancer cells compared to normal cells. Moreover, it also suggests that METS activates extrinsic and intrinsic apoptosis in oral cancer cells since caspases 8 and 9 are activated. Furthermore, these apoptosis inductions were suppressed by NAC (Figure 6). Hence, METS promotes apoptosis of oral cancer cells relying on oxidative stress.

### 4.4. METS Preferentially Provokes DNA Damage to Oral Cancer Cells

Several natural products use the anticancer strategy by targeting DNA damage responses, such as γH2AX expression [59]. 8-OHdG is a typical oxidative-stress-dependent DNA damage [60]. Since METS induces oxidative stress, the DNA damage status warrants a detailed assessment. Using flow cytometry, the γH2AX and 8-OHdG DNA damage were caused after METS treatment for oral cancer, but they showed lower levels in normal cells (Figure 9 and Figure 10). Moreover, these DNA damage inductions were suppressed by NAC. Accordingly, METS preferentially provokes ROS-dependent DNA damage to oral cancer cells, solidly impacting preferential antiproliferation.

### 4.5. METS Preferentially Arrests the Cell Cycle in Oral Cancer Cells

Marine sponge extracts disturb cell cycle progression in cancer cells. For example, petroleum ether extract of *Negombata magnifica* induces G1 arrest in liver cancer cells [57]. Methanol extract of *Crambe crambe* causes G2/M arrest in prostate cancer cells [61]. In addition to subG1 accumulation, METS caused G1 blocking of oral cancer Ca9-22 cells and G2/M blocking of oral cancer CAL 27 and normal S-G cells. Consequently, METS induced different responses to cell cycle disturbance in different oral cancer cell lines. Therefore, different marine sponge extracts may block the cell cycle progression at different phases in different cancer cells.

## 5. Conclusions

This study confirms that the antiproliferation effects of METS are effective in oral cancer cells but do not affect normal cells. This METS-induced preferential antiproliferation effects is associated with higher expressions of cellular and mitochondrial oxidative stress (ROS and mitoSOX), apoptosis (subG1 accumulation, annexin V enhancement, and upregulation of the extrinsic and intrinsic caspase signaling), and DNA damage (DNA double-strand breaks and oxidative damage) in breast cancer cells than in normal cells. Utilizing NAC pretreatment demonstrates that preferential antiproliferation function and mechanism of METS provides an oxidative-stress-mediated regulation. In conclusion, METS is a promising marine-sponge-derived natural product for antioral cancer treatment.

## Data Availability

Data are contained within the article.

## References

[1] Petersen P.E. (2009). Oral cancer prevention and control--the approach of the World Health Organization. Oral Oncol..

[2] Montero P.H., Patel S.G. (2015). Cancer of the oral cavity. Surg. Oncol. Clin. North Am..

[3] Silverman S. (1999). Oral cancer: Complications of therapy. Oral Surg. Oral Med. Oral Pathol. Oral Radiol. Endod..

[4] Singh R., Sharma M., Joshi P., Rawat D.S. (2008). Clinical status of anti-cancer agents derived from marine sources. Anticancer. Agents Med. Chem..

[5] Sithranga Boopathy N., Kathiresan K. (2010). Anticancer drugs from marine flora: An overview. J. Oncol..

[6] Farooqi A.A., Fayyaz S., Hou M.F., Li K.T., Tang J.Y., Chang H.W. (2014). Reactive oxygen species and autophagy modulation in non-marine drugs and marine drugs. Mar. Drugs.

[7] Lee M.G., Liu Y.C., Lee Y.L., El-Shazly M., Lai K.H., Shih S.P., Ke S.C., Hong M.C., Du Y.C., Yang J.C. (2018). Heteronemin, a marine sesterterpenoid-type metabolite, induces apoptosis in prostate LNcap cells via oxidative and ER stress combined with the inhibition of topoisomerase II and Hsp90. Mar. Drugs.

[8] Mehbub M.F., Lei J., Franco C., Zhang W. (2014). Marine sponge derived natural products between 2001 and 2010: Trends and opportunities for discovery of bioactives. Mar. Drugs.

[9] Mehbub M.F., Perkins M.V., Zhang W., Franco C.M.M. (2016). New marine natural products from sponges (Porifera) of the order Dictyoceratida (2001 to 2012); a promising source for drug discovery, exploration and future prospects. Biotechnol. Adv..

[10] Calcabrini C., Catanzaro E., Bishayee A., Turrini E., Fimognari C. (2017). Marine sponge natural products with anticancer potential: An updated review. Mar. Drugs.

[11] Varijakzhan D., Loh J.Y., Yap W.S., Yusoff K., Seboussi R., Lim S.E., Lai K.S., Chong C.M. (2021). Bioactive compounds from marine sponges: Fundamentals and applications. Mar. Drugs.

[12] Perdicaris S., Vlachogianni T., Valavanidis A. (2013). Bioactive natural substances from marine sponges: New developments and prospects for future pharmaceuticals. Nat. Prod. Chem. Res..

[13] Warsidah M., Sofiana M.S.J., Safitri I., Sapar A., Aritonang A.B., Muttalib Y., Fadly D. (2020). Protein isolation from sponge *Niphates* sp. as an antibacterial and antioxidant. Syst. Rev. Pharm..

[14] Kumar M.S., Vijayalaxmi K., Pal A. (2014). Antiinflamatuvar and antioxidant properties of *Spongosorites halichondriodes*, a marine sponge. Turk. J. Pharm. Sci..

[15] De Marino S., Ummarino R., D’Auria M.V., Chini M.G., Bifulco G., Renga B., D’Amore C., Fiorucci S., Debitus C., Zampella A. (2011). Theonellasterols and conicasterols from *Theonella swinhoei*. Novel marine natural ligands for human nuclear receptors. J. Med. Chem..

[16] Oiki S., Muramatsu I., Matsunaga S., Fusetani N. (1997). A channel-forming peptide toxin: Polytheonamide from marine sponge (*Theonella swinhoei*). Nihon Yakurigaku Zasshi.

[17] De Marino S., Festa C., D’Auria M.V., Cresteil T., Debitus C., Zampella A. (2011). Swinholide J, a potent cytotoxin from the marine sponge *Theonella swinhoei*. Mar. Drugs.

[18] Sinisi A., Calcinai B., Cerrano C., Dien H.A., Zampella A., D’Amore C., Renga B., Fiorucci S., Taglialatela-Scafati O. (2013). New tridecapeptides of the theonellapeptolide family from the Indonesian sponge *Theonella swinhoei*. Beilstein J. Org. Chem..

[19] Youssef D.T., Shaala L.A., Mohamed G.A., Badr J.M., Bamanie F.H., Ibrahim S.R. (2014). Theonellamide G, a potent antifungal and cytotoxic bicyclic glycopeptide from the Red Sea marine sponge *Theonella swinhoei*. Mar. Drugs.

[20] Fukuhara K., Takada K., Okada S., Matsunaga S. (2015). Nazumazoles A-C, cyclic pentapeptides dimerized through a disulfide bond from the marine sponge *Theonella swinhoei*. Org. Lett..

[21] Issac M., Aknin M., Gauvin-Bialecki A., De Voogd N., Ledoux A., Frederich M., Kashman Y., Carmeli S. (2017). Cyclotheonellazoles A-C, potent protease inhibitors from the marine sponge *Theonella* aff. swinhoei. J. Nat. Prod..

[22] Yang F., Li Y.Y., Tang J., Sun F., Lin H.W. (2018). New 4-methylidene sterols from the marine sponge *Theonella swinhoei*. Fitoterapia.

[23] Lai K.H., Peng B.R., Su C.H., El-Shazly M., Sun Y.L., Shih M.C., Huang Y.T., Yen P.T., Wang L.S., Su J.H. (2021). Anti-proliferative potential of secondary metabolites from the marine sponge *Theonella* sp.: Moving from correlation toward causation. Metabolites.

[24] Kumar M.S., Adki K.M. (2018). Marine natural products for multi-targeted cancer treatment: A future insight. Biomed. Pharmacother..

[25] Koeberle A., Werz O. (2014). Multi-target approach for natural products in inflammation. Drug Discov. Today.

[26] Chamberlin S.R., Blucher A., Wu G., Shinto L., Choonoo G., Kulesz-Martin M., McWeeney S. (2019). Natural product target network reveals potential for cancer combination therapies. Front. Pharmacol..

[27] Mioso R., Marante F.J., Bezerra R.S., Borges F.V., Santos B.V., Laguna I.H. (2017). Cytotoxic compounds derived from marine sponges. A review (2010–2012). Molecules.

[28] Kasten F.H., Pineda L.F., Schneider P.E., Rawls H.R., Foster T.A. (1989). Biocompatibility testing of an experimental fluoride releasing resin using human gingival epithelial cells *in vitro*. Vitro Cell Dev. Biol..

[29] Kasten F.H., Soileau K., Meffert R.M. (1990). Quantitative evaluation of human gingival epithelial cell attachment to implant surfaces *in vitro*. Int. J. Periodontics Restor. Dent..

[30] Hsieh P.L., Liao Y.W., Hsieh C.W., Chen P.N., Yu C.C. (2020). Soy isoflavone genistein impedes cancer stemness and mesenchymal transition in head and neck cancer through activating miR-34a/RTCB axis. Nutrients.

[31] Wang H.R., Tang J.Y., Wang Y.Y., Farooqi A.A., Yen C.Y., Yuan S.F., Huang H.W., Chang H.W. (2019). Manoalide preferentially provides antiproliferation of oral cancer cells by oxidative stress-mediated apoptosis and DNA damage. Cancers.

[32] Hung J.H., Chen C.Y., Omar H.A., Huang K.Y., Tsao C.C., Chiu C.C., Chen Y.L., Chen P.H., Teng Y.N. (2016). Reactive oxygen species mediate Terbufos-induced apoptosis in mouse testicular cell lines via the modulation of cell cycle and pro-apoptotic proteins. Environ. Toxicol..

[33] Huang C.H., Yeh J.M., Chan W.H. (2018). Hazardous impacts of silver nanoparticles on mouse oocyte maturation and fertilization and fetal development through induction of apoptotic processes. Environ. Toxicol..

[34] Wu C.F., Lee M.G., El-Shazly M., Lai K.H., Ke S.C., Su C.W., Shih S.P., Sung P.J., Hong M.C., Wen Z.H. (2018). Isoaaptamine induces T-47D cells apoptosis and autophagy via oxidative stress. Mar. Drugs.

[35] Yeh C.C., Tseng C.N., Yang J.I., Huang H.W., Fang Y., Tang J.Y., Chang F.R., Chang H.W. (2012). Antiproliferation and induction of apoptosis in Ca9-22 oral cancer cells by ethanolic extract of *Gracilaria tenuistipitata*. Molecules.

[36] Vignon C., Debeissat C., Georget M.T., Bouscary D., Gyan E., Rosset P., Herault O. (2013). Flow cytometric quantification of all phases of the cell cycle and apoptosis in a two-color fluorescence plot. PLoS ONE.

[37] Fan H.C., Hsieh Y.C., Li L.H., Chang C.C., Janouskova K., Ramani M.V., Subbaraju G.V., Cheng K.T., Chang C.C. (2020). Dehydroxyhispolon methyl ether, a hispolon derivative, inhibits WNT/beta-catenin signaling to elicit human colorectal carcinoma cell apoptosis. Int. J. Mol. Sci..

[38] Liu W., Lin L.C., Wang P.J., Chen Y.N., Wang S.C., Chuang Y.T., Tsai I.H., Yu S.Y., Chang F.R., Cheng Y.B. (2021). Nepenthes ethyl acetate extract provides oxidative stress-dependent anti-leukemia effects. Antioxidants.

[39] Lin C.H., Chan H.S., Tsay H.S., Funayama S., Kuo C.L., Chung J.G. (2018). Ethyl acetate fraction from methanol extraction of *Vitis thunbergii* var. taiwaniana induced G0/G1 phase arrest via inhibition of cyclins D and E and induction of apoptosis through caspase-dependent and -independent pathways in human prostate carcinoma DU145 cells. Environ. Toxicol..

[40] Liu S.L., Yang K.H., Yang C.W., Lee M.Y., Chuang Y.T., Chen Y.N., Chang F.R., Chen C.Y., Chang H.W. (2021). Burmannic acid inhibits proliferation and induces oxidative stress response of oral cancer cells. Antioxidants.

[41] Shiau J.P., Chuang Y.T., Yang K.H., Chang F.R., Sheu J.H., Hou M.F., Jeng J.H., Tang J.Y., Chang H.W. (2022). Brown algae-derived fucoidan exerts oxidative stress-dependent antiproliferation on oral cancer cells. Antioxidants.

[42] Chiu C.C., Huang J.W., Chang F.R., Huang K.J., Huang H.M., Huang H.W., Chou C.K., Wu Y.C., Chang H.W. (2013). Golden berry-derived 4beta-hydroxywithanolide E for selectively killing oral cancer cells by generating ROS, DNA damage, and apoptotic pathways. PLoS ONE.

[43] El-Damhougy K., El-Naggar H.A., Ibrahim H., Bashar M.A., Abou-Senna F.M. (2017). Biological activities of some marine sponge extracts from Aqaba Gulf, Red Sea, Egypt. Int. J. Fish. Aquat. Stud..

[44] Choi C., Son A., Lee H.S., Lee Y.J., Park H.C. (2018). Radiosensitization by marine sponge *Agelas* sp. extracts in hepatocellular carcinoma cells with autophagy induction. Sci. Rep..

[45] Ciftci H.I., Can M., Ellakwa D.E., Suner S.C., Ibrahim M.A., Oral A., Sekeroglu N., Ozalp B., Otsuka M., Fujita M. (2020). Anticancer activity of Turkish marine extracts: A purple sponge extract induces apoptosis with multitarget kinase inhibition activity. Invest New Drugs.

[46] Lin A. (2018). Radiation therapy for oral cavity and oropharyngeal cancers. Dent. Clin. North Am..

[47] Hartner L. (2018). Chemotherapy for oral cancer. Dent. Clin. North Am..

[48] Acharya A., Das I., Chandhok D., Saha T. (2010). Redox regulation in cancer: A double-edged sword with therapeutic potential. Oxid. Med. Cell Longev..

[49] Tang J.Y., Ou-Yang F., Hou M.F., Huang H.W., Wang H.R., Li K.T., Fayyaz S., Shu C.W., Chang H.W. (2019). Oxidative stress-modulating drugs have preferential anticancer effects—Involving the regulation of apoptosis, DNA damage, endoplasmic reticulum stress, autophagy, metabolism, and migration. Semin. Cancer Biol..

[50] Gorrini C., Harris I.S., Mak T.W. (2013). Modulation of oxidative stress as an anticancer strategy. Nat. Rev. Drug Discov..

[51] Kim S.J., Kim H.S., Seo Y.R. (2019). Understanding of ROS-inducing strategy in anticancer therapy. Oxid Med. Cell Longev..

[52] Sies H. (2019). Oxidative stress: Eustress and distress in redox homeostasis. Stress: Physiology, Biochemistry, and Pathology.

[53] Ahmad T., Suzuki Y.J. (2019). Juglone in oxidative stress and cell signaling. Antioxidants.

[54] Li R., Huang C., Ho J.C.H., Leung C.C.T., Kong R.Y.C., Li Y., Liang X., Lai K.P., Tse W.K.F. (2021). The use of glutathione to reduce oxidative stress status and its potential for modifying the extracellular matrix organization in cleft lip. Free Radic. Biol. Med..

[55] Abboud M.M., Al Awaida W., Alkhateeb H.H., Abu-Ayyad A.N. (2019). Antitumor action of amygdalin on human breast cancer cells by selective sensitization to oxidative stress. Nutr. Cancer.

[56] Zou Z., Chang H., Li H., Wang S. (2017). Induction of reactive oxygen species: An emerging approach for cancer therapy. Apoptosis.

[57] Rady H.M., Hassan A.Z., Salem S.M., Mohamed T.K., Esmaiel N.N., Ez-El-Arab M.A., Ibrahim M.A., Fouda F.K. (2016). Induction of apoptosis and cell cycle arrest by *Negombata magnifica* sponge in hepatocellular carcinoma. Med. Chem. Res..

[58] Annamalai P., Thayman M., Rajan S., Raman L.S., Ramasubbu S., Perumal P. (2015). Ethyl acetate extract from marine sponge *Hyattella cribriformis* exhibit potent anticancer activity by promoting tubulin polymerization as evidenced mitotic arrest and induction of apoptosis. Pharmacogn. Mag..

[59] Van Stuijvenberg J., Proksch P., Fritz G. (2020). Targeting the DNA damage response (DDR) by natural compounds. Bioorg. Med. Chem..

[60] Arfin S., Jha N.K., Jha S.K., Kesari K.K., Ruokolainen J., Roychoudhury S., Rathi B., Kumar D. (2021). Oxidative stress in cancer cell metabolism. Antioxidants.

[61] Ottinger S., Kloppel A., Rausch V., Liu L., Kallifatidis G., Gross W., Gebhard M.M., Brummer F., Herr I. (2012). Targeting of pancreatic and prostate cancer stem cell characteristics by *Crambe crambe* marine sponge extract. Int. J. Cancer.

